# Decades Later: Recurrent Gastrointestinal Sequelae in a Patient With Remote Metastatic Osteosarcoma to the Stomach and Colon

**DOI:** 10.7759/cureus.115681

**Published:** 2026-09-02

**Authors:** Adriana Gurreri, Alvaro Parra Ayala, Ivana Rubenstein

**Affiliations:** 1 Internal Medicine, Nova Southeastern University Dr. Kiran C. Patel College of Osteopathic Medicine, Davie, USA; 2 Internal Medicine, Broward Health Medical Center, Fort Lauderdale, USA

**Keywords:** abdominal pain secondary to osteosarcoma, intra-abdominal metastasis, metastatic osteosarcoma, recurrent osteosarcoma, unusual metastasis

## Abstract

Osteosarcoma is a primary malignant bone tumor with a well-established tendency for pulmonary metastasis; however, gastrointestinal (GI) involvement is exceedingly rare. Intra-abdominal metastases may occur years to decades after the initial diagnosis. Hematogenous spread, following pulmonary metastasis, is the presumed mechanism. Due to delayed presentation and atypical manifestations, recognition of GI complications in patients with a remote history of osteosarcoma is essential. In this report, we present a case of an 80-year-old male with chronic GI complications decades after original metastatic osteosarcoma diagnosis involving the stomach and colon. He described recurrent, dull, constant left lower quadrant abdominal pain associated with poor oral intake and constipation. Physical examination demonstrated focal left lower quadrant tenderness. CT abdomen and pelvis revealed dilated small bowel loops concerning for partial small bowel obstruction, while esophagram demonstrated esophageal dysmotility with mild reflux. These findings are linked to the sequelae of complications following initial metastatic osteosarcoma. The patient had a history of numerous surgeries and required continuous opioid use for pain management. Conservative management consisted of bowel rest, laxatives, and supportive care resulting in transient symptom improvement; however, symptoms recurred frequently following discharge, leading to multiple readmissions for recurrent obstructive and dysmotility-related symptoms. This case highlights several gastroenterological considerations. Metastatic osteosarcoma and further GI sequelae should remain within the differential diagnoses of chronic abdominal pain or bowel obstruction in patients with a remote history of osteosarcoma, even decades after initial treatment. For gastroenterologists, recognition of both structural and functional GI sequelae of osteosarcoma is critical to guide symptom-directed, nonoperative management strategies and reduce recurrent hospitalizations.

## Introduction

Osteosarcoma is a primary bone cancer involving the diagnosis of a malignant bone tumor with an annual incidence of three to five cases per million people in the USA [1]. Management involves treatment of primary tumors and consideration of the metastatic potential identified with the disease, most prominently with lung metastases [2]. In comparison to the common nature of lung metastases, intra-abdominal metastasis is incredibly unusual [2]. Few existing studies have reported cases involving the metastasis of osteosarcoma to the colon [3,4]. A study reviewing osteosarcoma metastasis in 94 osteosarcoma cases identified two involved abdominal metastases, and both were after lung metastasis was previously identified [5]. In these cases, the patient’s abdominal metastases were found more than three years after their initial diagnosis [5]. This reinforces the importance of continued monitoring years after initial diagnosis. It is presumed that intra-abdominal metastasis occurs via the hematogenous route, most likely from a prior pulmonary metastasis. Most intra-abdominal metastases cited in the literature present as solid masses, and therefore any abdominal mass should warrant further investigation in patients with a history of osteosarcoma [5]. 

For therapeutic reasons, it is important to consider osteosarcoma as a systemic disease with robust potential for spread beyond the primary site of diagnosis; therefore, thorough investigation for metastasis is essential [1,2,6]. In this report, we present a notable case given the rarity of this condition involving osteosarcoma metastasis to the colon and stomach in coordination with other chronic complications.

## Case presentation

The patient is an 80-year-old male with a history of left distal femoral osteosarcoma status post above-the-knee amputation at age 30, metastasis to left lower lobe of the lung status post resection at age 40, metastasis to stomach and colon status post resection at age 71 and insertion of an abdominal morphine pump presenting with abdominal pain and dysphasia. His symptoms are constant, but he recently experienced an exacerbation. He presented with recurrent, dull, and constant pain most severe in the left lower quadrant of the abdomen associated with poor oral intake and constipation. He endorses passing gas and denies nausea, vomiting, and fevers. 

Physical exam on admission showed normal bowel sounds, soft abdomen, focal abdominal tenderness in the left lower quadrant, superficial right lower quadrant morphine pump, no abdominal distension, and above-the-knee left lower extremity amputation. The rest of the physical exam was unremarkable. 

CT abdomen and pelvis in the emergency room show focally dilated loops of small bowel in the right lower quadrant suggestive of partial small bowel obstruction, as seen in Figure 1. General surgery was consulted and recommended a nasogastric tube but the patient declined it. He was given bisacodyl, senna-docusate, IV morphine and instructed to refrain from abdominal morphine pump while hospitalized. Two days later, as displayed in Figure 2, an upper gastrointestinal (GI) series with small bowel follow-through showed no evidence of small bowel obstruction. An esophagram shown in Figure 3 was then performed and showed esophageal disordered motility in the distal thoracic esophagus with mild reflux, but no evidence of small bowel obstruction. His symptoms transiently improved with conservative management including bowel rest, laxatives, and supportive care within three days, and he was subsequently discharged. However, during the next few months, he has had recurrent monthly admissions for similar complaints including recurrent obstructive and dysmotility-related symptoms that are treated similarly. 

**Figure 1:**
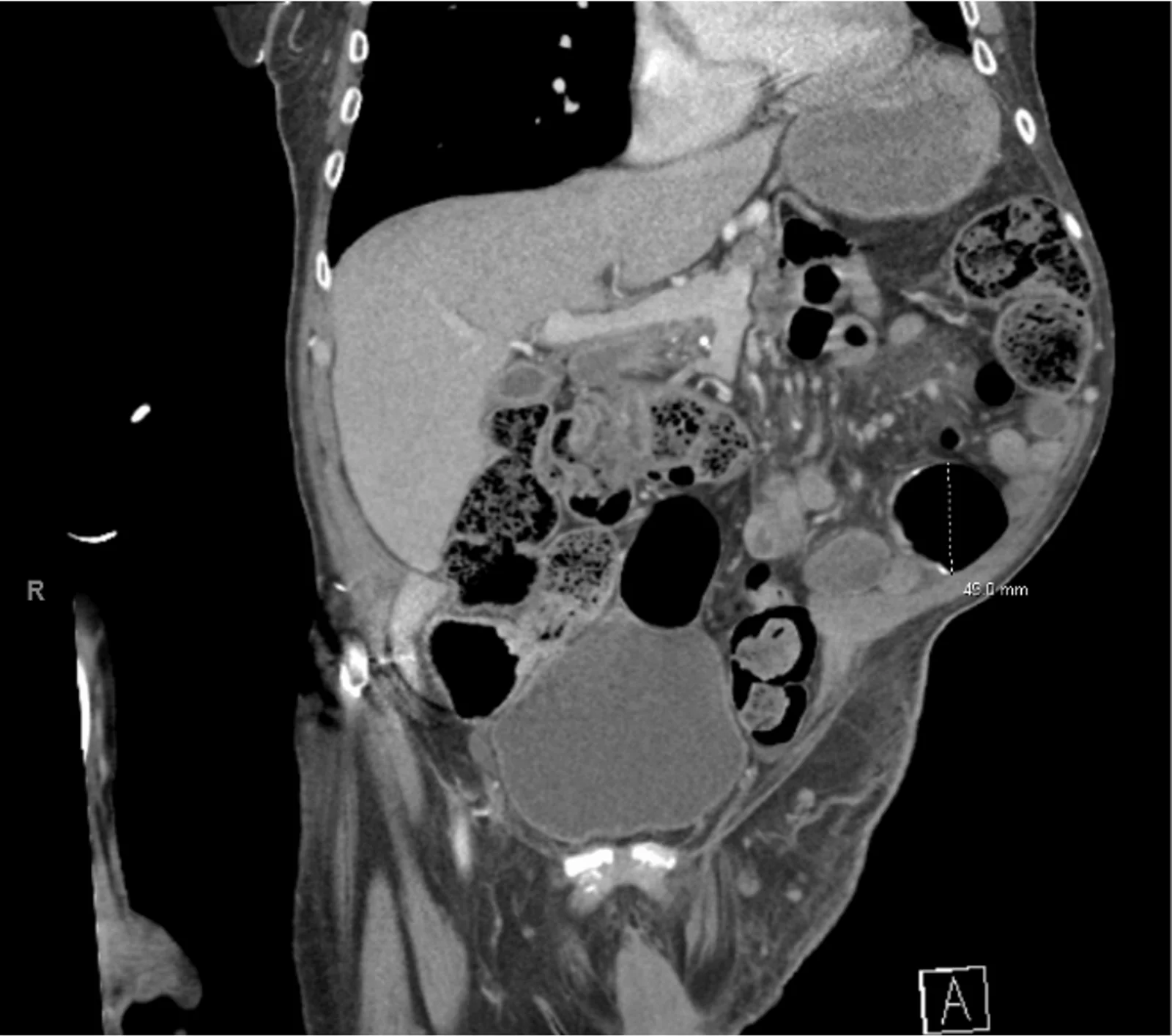
Coronal CT image of the abdomen and pelvis showing focally dilated small-bowel loops in the right lower quadrant, concerning for partial small-bowel obstruction

**Figure 2:**
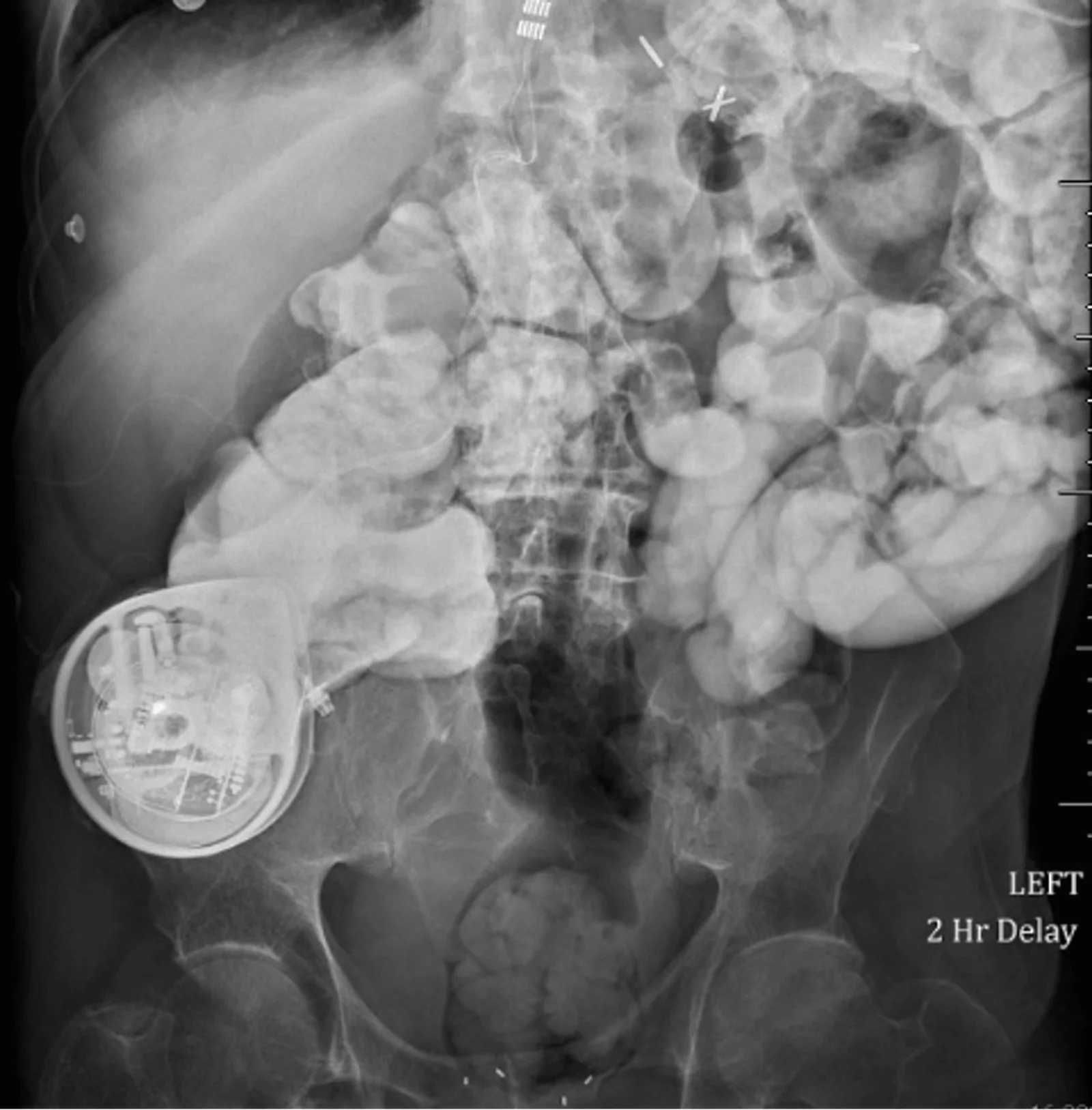
Upper Gastrointestinal (GI) Series with small bowel follow-through demonstrating no evidence of small bowel obstruction

**Figure 3:**
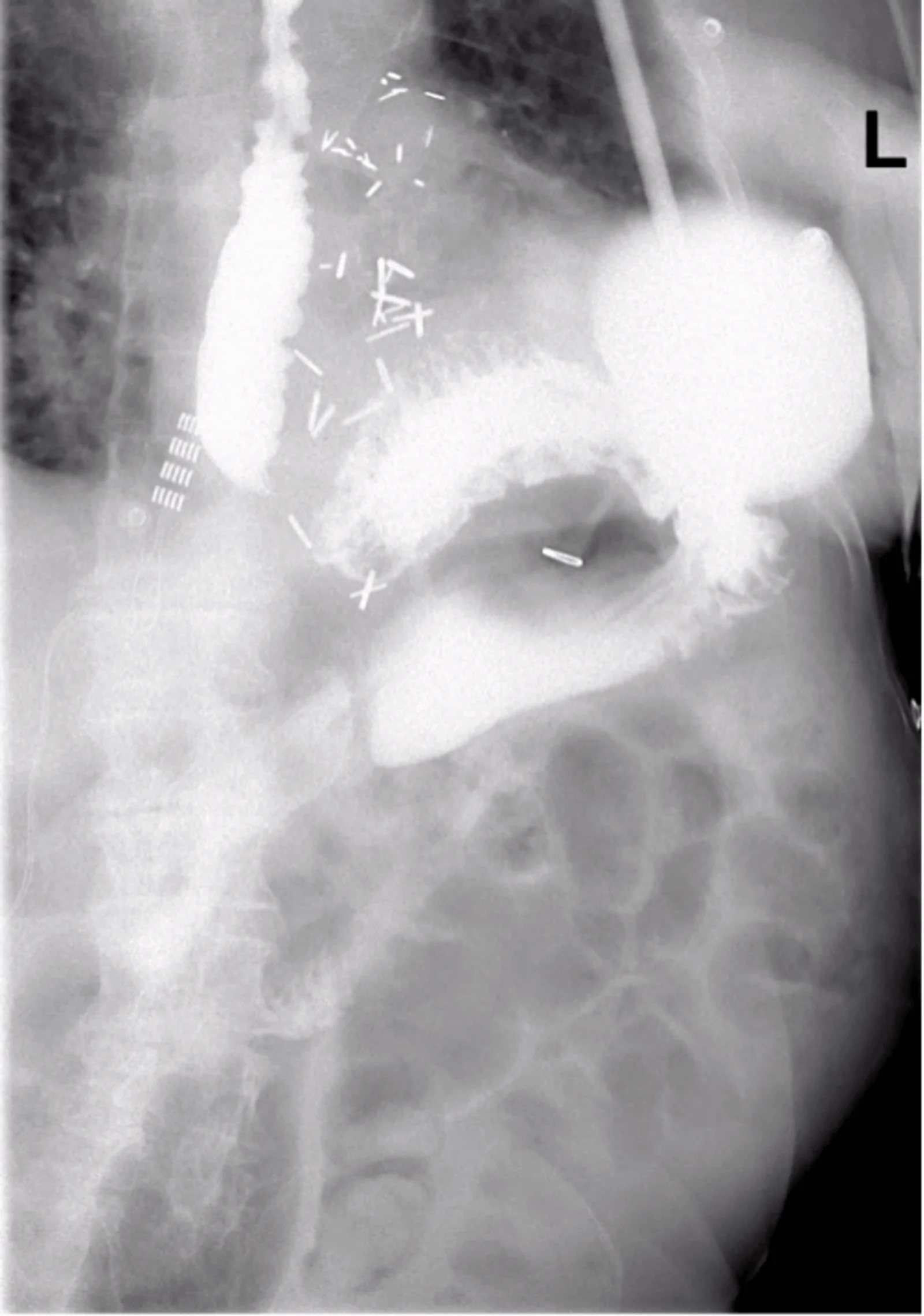
Esophagram demonstrating esophageal dysmotility in the distal thoracic esophagus with mild reflux, but no evidence of small bowel obstruction

## Discussion

This case highlights several gastroenterological considerations. Metastatic osteosarcoma should remain within the differential diagnoses of chronic abdominal pain or bowel obstruction in patients with a remote history of osteosarcoma [7]. Relapse can occur decades later, and osteosarcoma relapse more than 10 years following initial diagnosis is associated with increased complications, poor prognosis and reduced quality of life [8]. The presentation of osteosarcoma metastasis to the abdomen in this case is an unusual route of metastasis. In the literature, this atypical spread to the abdomen has previously been associated with prior lung metastases as seen in this case. GI morbidity in these patients is often multifactorial, resulting from postoperative adhesions, altered bowel anatomy, and chronic dysmotility. Increased complications and comorbid impediments resulting from primary disease are associated with recurrent hospitalizations. As displayed in this case, prior resections of the colon and possible postoperative adhesions may result in intermittent obstruction. Bowel adhesions can be a response to surgery in attempts of innate repair that may result in obstruction [9]. The patient’s initial osteosarcoma diagnosis followed by eventual metastasis to the abdomen resulted in chronic pain and required use of a morphine pump. This can lead to associated adverse effects, including constipation and recurrent small bowel obstructions due to GI motility delays and result in increased episodes of pain and frequent readmissions. Early interventions and prolonged follow-up in patients with an osteosarcoma diagnosis can assist in improving future quality of life and comorbidities [7]. 

## Conclusions

Thoughtful consideration of metastatic potential and complications in patients with a history of osteosarcoma is essential in care and management. For gastroenterologists, recognition of both structural and functional GI sequelae of osteosarcoma is critical. Clinicians can carefully consider identifying the indications for further surveillance imaging, motility evaluation and nutritional support if needed. Furthermore, encouraging interdisciplinary care coordination through follow-up adherence to oncology, palliative care for symptom control, and pain medicine for opioid-minimizing pain management. This approach allows for guidance of symptom-directed, nonoperative management strategies and reduction of recurrent hospitalizations. 
